# Supplementary material for: Synthetic electronic health records generated with variational graph autoencoders
Source: NPJ Digit Med. 2023 Apr 29;6:83. doi: 10.1038/s41746-023-00822-x (PMC10148837; doi:10.1038/s41746-023-00822-x)
Supplement: Supplementary file 1 — REPORTING SUMMARY [file 41746_2023_822_MOESM1_ESM.pdf]

nature portfolio

Corresponding author(s): Giannis Nikolentzos
Last updated by author(s): Feb 24, 2023

Reporting Summary

Nature Portfolio wishes to improve the reproducibility of the work that we publish. This form provides structure for consistency and transparency in reporting. For further information on Nature Portfolio policies, see our Editorial Policies and the Editorial Policy Checklist.

Statistics

For all statistical analyses, confirm that the following items are present in the figure legend, table legend, main text, or Methods section.

n/a	Confirmed	
☐	☒	The exact sample size (*n*) for each experimental group/condition, given as a discrete number and unit of measurement
☒	☐	A statement on whether measurements were taken from distinct samples or whether the same sample was measured repeatedly
☒	☐	The statistical test(s) used AND whether they are one- or two-sided<br>*Only common tests should be described solely by name; describe more complex techniques in the Methods section.*
☒	☐	A description of all covariates tested
☒	☐	A description of any assumptions or corrections, such as tests of normality and adjustment for multiple comparisons
☐	☒	A full description of the statistical parameters including central tendency (e.g. means) or other basic estimates (e.g. regression coefficient) AND variation (e.g. standard deviation) or associated estimates of uncertainty (e.g. confidence intervals)
☒	☐	For null hypothesis testing, the test statistic (e.g. *F*, *t*, *r*) with confidence intervals, effect sizes, degrees of freedom and *P* value noted<br>*Give P values as exact values whenever suitable.*
☒	☐	For Bayesian analysis, information on the choice of priors and Markov chain Monte Carlo settings
☒	☐	For hierarchical and complex designs, identification of the appropriate level for tests and full reporting of outcomes
☒	☐	Estimates of effect sizes (e.g. Cohen's *d*, Pearson's *r*), indicating how they were calculated

Our web collection on statistics for biologists contains articles on many of the points above.

Software and code

Policy information about availability of computer code

Data collection

The MIMIC-IV database§ was imported into the SHAARPEC Analytics platform§§. Patient trajectories were extracted as graphs from the SHAARPEC Analytics API. The construction of the patient trajectories are described in the manuscript. The (raw) extracted patient trajectories are available to the editors and reviewers on request.

§ Johnson, A.E.W., Bulgarelli, L., Shen, L. et al. MIMIC-IV, a freely accessible electronic health record dataset. Sci Data 10, 1 (2023). https://doi.org/10.1038/s41597-022-01899-x
§§ https://api-demo.shaarpec.com

Data analysis

The patient graphs were processed and analyzed in Jupyter notebooks, using the Python open-source data science ecosystem. The deep learning model was built and analyzed with pytorch 1.12.1, pandas 1.5.2, networkx 3.0, seaborn 0.12.1, scikit-learn 1.1.3 and grakel 0.1.9.

For manuscripts utilizing custom algorithms or software that are central to the research but not yet described in published literature, software must be made available to editors and reviewers. We strongly encourage code deposition in a community repository (e.g. GitHub). See the Nature Portfolio guidelines for submitting code & software for further information.

Data

Policy information about availability of data

All manuscripts must include a data availability statement. This statement should provide the following information, where applicable:

- Accession codes, unique identifiers, or web links for publicly available datasets
- A description of any restrictions on data availability
- For clinical datasets or third party data, please ensure that the statement adheres to our policy

The Medical Information Mart for Intensive Care (MIMIC-IV) database was the source to all our numerical experiments§. MIMIC-IV provides critical care data for thousands of patients admitted to the intensive care units at the Beth Israel Deaconess Medical Center. Individuals interested in accessing MIMIC-IV must complete a training course in research with human participants and sign a data use agreement (DUA). The DUA requires users to adequately safeguard the dataset, to not attempt to reidentify individuals, to not share the data, and to report issues relating to deidentification.

§ Johnson, A.E.W., Bulgarelli, L., Shen, L. et al. MIMIC-IV, a freely accessible electronic health record dataset. Sci Data 10, 1 (2023). https://doi.org/10.1038/s41597-022-01899-x

Human research participants

Policy information about studies involving human research participants and Sex and Gender in Research.

Reporting on sex and gender	The MIMIC-IV source data makes no distinction between ethnicity and gender information, and is diverse and inclusive. There is no reporting on gender specific data.
Population characteristics	See above.
Recruitment	The data was collected from the emergency unit and in-patient stays at Beth Israel Deaconess Medical Center (BIDMC) in Boston, MA as described in Johnson, A.E.W., Bulgarelli, L., Shen, L. et al. MIMIC-IV, a freely accessible electronic health record dataset. Sci Data 10, 1 (2023). https://doi.org/10.1038/s41597-022-01899-x.
Ethics oversight	The creation of the MIMIC-IV database was approved by the Institutional Review Boards of Beth Israel Deaconess Medical Center (Boston, MA) and the Massachusetts Institute of Technology (Cambridge, MA), as described in Johnson et al.§ and the related publications. Requirement for individual patient consent was waived because the project did not impact clinical care and all protected health information was deidentified. § Johnson, A.E.W., Bulgarelli, L., Shen, L. et al. MIMIC-IV, a freely accessible electronic health record dataset. Sci Data 10, 1 (2023). https://doi.org/10.1038/s41597-022-01899-x

Note that full information on the approval of the study protocol must also be provided in the manuscript.

Field-specific reporting

Please select the one below that is the best fit for your research. If you are not sure, read the appropriate sections before making your selection.

☒ Life sciences ☐ Behavioural & social sciences ☐ Ecological, evolutionary & environmental sciences

For a reference copy of the document with all sections, see nature.com/documents/nr-reporting-summary-flat.pdf

Life sciences study design

All studies must disclose on these points even when the disclosure is negative.

Sample size	The sample size was determined by the atrial fibrillation cohort in the MIMIC-IV database (~6500 patients). This sample size is considerally larger than a typical clinical trial (~1000 patients).
Data exclusions	Data points (i.e., diagnosis and medication codes) that occured less than 50 times in the total in the data were excluded. The model will struggle to learn such rare features, and they are so infrequent that they do not contribute to the distribution properties of interest in this study.
Replication	Not applicable since this is not an in-vitro/in-vivo/clinical trial study.
Randomization	Not applicable since this is not an in-vitro/in-vivo/clinical trial study.
Blinding	Not applicable since this is not an in-vitro/in-vivo/clinical trial study.

Reporting for specific materials, systems and methods

We require information from authors about some types of materials, experimental systems and methods used in many studies. Here, indicate whether each material, system or method listed is relevant to your study. If you are not sure if a list item applies to your research, read the appropriate section before selecting a response.

Materials & experimental systems

n/a	Involved in the study
☒	☐ Antibodies
☒	☐ Eukaryotic cell lines
☒	☐ Palaeontology and archaeology
☒	☐ Animals and other organisms
☐	☒ Clinical data
☒	☐ Dual use research of concern

Methods

n/a	Involved in the study
☒	☐ ChIP-seq
☒	☐ Flow cytometry
☒	☐ MRI-based neuroimaging

Clinical data

Policy information about clinical studies

All manuscripts should comply with the ICMJE guidelines for publication of clinical research and a completed CONSORT checklist must be included with all submissions.

Clinical trial registration	Not applicable since this is not an in-vitro/in-vivo/clinical trial study.
Study protocol	Not applicable since this is not an in-vitro/in-vivo/clinical trial study.
Data collection	Not applicable since this is not an in-vitro/in-vivo/clinical trial study.
Outcomes	Not applicable since this is not an in-vitro/in-vivo/clinical trial study.
