# Supplementary material for: Synthetic electronic health records generated with variational graph autoencoders
Source: NPJ Digit Med. 2023 Apr 29;6:83. doi: 10.1038/s41746-023-00822-x (PMC10148837; doi:10.1038/s41746-023-00822-x)
Supplement: Supplementary file 2 — Supplementary Table 5 [file 41746_2023_822_MOESM2_ESM.pdf]

Table 5: **Classification accuracy on the test data set in the task of distinguishing between real and synthetic patient trajectories.** 6535 synthetic trajectories were generated to train a SVM classifier (together with equally many real trajectories) to distinguish between real and synthetic trajectories. Both Weisfeiler-Lehman (WL) and shortest path (SP) graph kernels were used. Two scenarios were investigated. Scenario 1 accounts only for graph structure while scenario 2 in addition considers node labels. The results showed that including node labels increased the classifier's prediction, indicating that some complex data patterns were more (or less) common in the synthetic data.

	Without Labels	With Labels
WL	58%	72%
SP	56%	73%
